# Drift and shape—new insights into human immunity against influenza virus neuraminidase

**DOI:** 10.1128/mbio.01654-23

**Published:** 2023-11-07

**Authors:** Annette Fox

**Affiliations:** 1 WHO Collaborating Centre for Reference and Research on Influenza, Royal Melbourne Hospital, at the Peter Doherty Institute for Infection and Immunity, Melbourne, Victoria, Australia; 2 Department of Infectious Diseases, University of Melbourne, at the Peter Doherty Institute for Infection and Immunity, Melbourne, Victoria, Australia; McMaster University, Hamilton, Canada

**Keywords:** influenza, humoral immunity, immune memory, neuraminidase, antigenic variation

## Abstract

Influenza virus hemagglutinin mediates infection by binding sialic acids, whereas neuraminidase cleaves sialic acids to release progeny virions. Both are targets of protective antibodies, but influenza vaccine strain selection and antigen dose are based on hemagglutinin alone. Virus characterization using first infection ferret sera indicates that escape from hemagglutination inhibiting (HI) antibodies occurs more frequently and is not coordinated with escape from neuraminidase inhibiting (NI) antibodies. A key question addressed by Daulagala et al. (P. Daulagala, B. R. Mann, K. Leung, E. H. Y. Lau, et al., mBio 14:e00084-23, 2023, https://doi.org/10.1128/mbio.00084-23) is how this translates to humans who encounter multiple influenza viruses throughout life. Their cross-sectional study, using sera from a wide age range of participants and H1N1 viruses spanning 1977–2015, indicates that NI antibodies are more broadly cross-reactive than HI antibodies. Both HI and NI titers were highest against strains encountered in childhood indicating that both are shaped by priming exposures. The study further supports the development of NA-optimized vaccines.

## COMMENTARY

Hemagglutinin (HA) and neuraminidase (NA) are the major targets of humoral immunity against influenza. HA exhibits the highest rates of genetic and antigenic evolution (1, 2) and is the target of immunity induced by influenza vaccines. Accordingly, the research community has spent decades investigating how exposure to successive variant influenza viruses shapes human antibody responses to HA. Although NA also exhibits substantial genetic evolution (1), there is limited understanding of how this shapes NA immunity and immune escape in humans. A call to better understand immunity to NA and how it can contribute to the design of better influenza vaccines specifically highlights the need for data on the breadth of the human NA response and the impact of pre-existing immunity (3). Some of the first data addressing these topics were presented recently in *mBio* (4). This commentary discusses key concepts related to this study including the mechanics of humoral immunity to influenza HA and NA, antigenic variation, and immunological imprinting.

Influenza viruses attach to host cell sialic acids through the HA protein. Antibodies inhibit virus attachment by binding sites in and around the sialic acid receptor binding pocket of HA. However, the combined effects of viral replication without proofreading and antibody selection pressure drive immune escape mutations in HA, otherwise termed antigenic drift (5). The strain composition of influenza vaccines is updated frequently based on genetic and antigenic characterization of circulating strain HAs. Nevertheless, influenza vaccine effectiveness can be poor, even when vaccine and circulating strains are well matched (6). This may in part be due to the effects of prior influenza exposures. Studies dating back to the 1950s indicate that HI antibody titers are higher against influenza viruses encountered during early life than against subsequently encountered viruses (7–9). This has led to theories that early life exposures imprint or shape responses to subsequently encountered strains through memory B cell recall (7, 10). Antibody responses can also become highly focused on limited epitopes that are conserved across successively encountered strains, indicating that memory B cell recall may limit *de novo* B cell responses against variant epitopes (11–13). Antibody and B cell responses can diminish with repeated influenza vaccination, particularly against strains that have not been updated, suggesting that existing antibodies may hinder B cell activation (14–16).

NA is a sialidase that cleaves HA-bound sialic acids allowing the release of newly formed virions (17, 18). Additionally, NA-mediated sialic acid cleavage helps viruses penetrate sialic acid-laden mucus (19). The NA catalytic site is a deep pocket comprising conserved amino acids in the head of NA (20). Rare antibodies with long heavy chain complementarity determining region 3 (CDR3) can inhibit the sialidase activity of NAs belonging to most subtypes by inserting the CDR3 loop into the catalytic site (21, 22). More commonly, NI antibodies bind epitopes nearby the catalytic site and sterically hinder interaction with sialic acids (19, 23). Some of the residues targeted by monoclonal antibodies (mAbs) with NA inhibiting (NI) activity against H1N1 readily mutate and may contribute to antigenic change (24–27). Other residues are relatively conserved and are recognized by mAbs that inhibit a broad range of strains (25, 28, 29). Several NI mAbs with activity against a range of N1 viruses were isolated from a patient with primary pandemic H1N1 infection, suggesting that exposure to a novel N1 favored recall of memory B cells generated against conserved epitopes (29). NI antibodies reduce virus plaque size (17), virus replication, symptoms, mortality, and transmission in animal models (21, 29–32) and correlate with protection against illness but not infection in humans (33–35). The NA content of inactivated influenza vaccines is incidental and maybe 3- to 200-fold lower than the HA content, which is fixed (36–38). Moreover, recombinant influenza vaccines only contain HA.

Studies that directly compare HA and NA genetic and antigenic evolution indicate that NA evolution has been slower and is not synchronized with HA evolution (24, 39, 40). In this issue of mBio, Daulagala et al. antigenically characterize NAs of H1N1 viruses circulating between 1977 and 1991 (4), complementing studies of viruses circulating between 1991 and 2017 (24, 25). Combined, these studies suggest that H1N1 viruses from 1977 to 2017 form six antigenically distinct NA clusters, whereas vaccine strains have been updated 11 times based on HA antigenic change (4, 24, 25, 41). The protective capacity of NI antibodies combined with the slower pace of NA antigenic evolution indicates that influenza vaccine effectiveness may be improved by optimizing the NA content. Substantial progress has been made toward this goal with multiple studies demonstrating that recombinant NA tetramers protect mice against weight loss and death in the case of lethal virus challenge (37, 42–44). Similarly, NA-based virus-like particles can protect ferrets from lethal avian influenza A (H5N1) infection (45), and mRNA vaccines encoding N1 or N2 NA protect mice against a range of viruses belonging to the same NA subtype (46, 47). Importantly, recombinant NA tetramers retain the ability to induce robust NI antibody titers when admixed with quadrivalent influenza vaccine if a toll-like-receptor agonist is included as an adjuvant (44). Further progress toward an NA-optimized vaccine requires clinical studies of these recombinant protein and mRNA formulations and may also benefit from structure-based protein designs that improve the conformational stability and immunogenicity of NA tetramers (48). There is also a need for data on human immunity to NA (3). A key question is whether NI antibodies are more broadly cross-reactive than HA-directed neutralizing or HI antibodies in humans and whether the breadth of activity is influenced by early-life influenza exposures, as found for HI antibodies.

Recently in *mBio*, Daulagala et al. examined the breadth of HI versus NI antibodies in the sera of humans born between 1950 and 2015 (4). Their study included 130 sera and 10 H1N1 viruses spanning 1977–2015. Titers of both HI and NI antibodies were highest against strains encountered in childhood. A previous study of antibody titers against three NAs representing H1N1 viruses from 1918, 1977, and 1999 shows similar age-related trends (49). It may be expected that memory will be induced against both HA and NA during priming infections and have similar effects on subsequent responses to drift variants even if they drift at different rates. However, for most of the H1N1 viruses tested, the age ranges of people who had detectable titers were greater for NI antibodies than for HI antibodies. Similarly, the proportion of participants who had cross-strain reactive antibodies was greater for NI than for HI antibodies. This cross-reactivity is consistent with the presence of NI antibodies that target relatively conserved epitopes (21, 25, 28, 29, 50, 51). Although NI mAbs that target conserved epitopes can protect across strains (21, 28, 29), primary antisera raised against NA can be relatively strain specific (25). It will be important to determine whether successive exposure to variant strains reinforces responses to conserved epitopes and promotes NI antibody breadth. One study found that while most recipients of inactivated influenza vaccine exhibit an NI titer rise, titers and titer rises were attenuated among repeatedly vaccinated participants (52). It is also thought that infectious viruses versus inactivated vaccines differ in their capacity to induce protective antibodies (51) due to the differences in NA structure (48). Therefore, studies that document exposure histories would be valuable.

The study by Daulagala et al. indicates that human NI antibodies exhibit greater cross-H1N1 strain activity than HI antibodies. Therefore, a vaccine that boosts NI antibodies may increase vaccine effectiveness by protecting against HA antigenic variants. Limited antigenic characterization indicates that H3N2 NA is more antigenically variable than H1N1 NA (27), and similar studies comparing the breadth of human HI and NI antibodies against H3N2 are needed. Further development of an NA-optimized vaccine is warranted and will require data on antibody titers required for protection and on the dose and form of NA required to induce these titers.
